# Prognostic nutritional index as a prognostic biomarker for gastrointestinal cancer patients treated with immune checkpoint inhibitors

**DOI:** 10.3389/fimmu.2023.1219929

**Published:** 2023-07-21

**Authors:** Lilong Zhang, Wangbin Ma, Zhendong Qiu, Tianrui Kuang, Kunpeng Wang, Baohong Hu, Weixing Wang

**Affiliations:** ^1^ Department of General Surgery, Renmin Hospital of Wuhan University, Wuhan, China; ^2^ Key Laboratory of Hubei Province for Digestive System Disease, Wuhan, China

**Keywords:** prognostic nutrition index, immune checkpoint inhibitors, gastrointestinal cancers, esophageal cancer, gastric cancer, hepatocellular carcinoma

## Abstract

**Objective:**

Our study represents the first meta-analysis conducted to evaluate the prognostic utility of the baseline prognostic nutritional index (PNI) in patients with gastrointestinal cancer (GIC) who received immune checkpoint inhibitor (ICI) therapy.

**Methods:**

We searched PubMed, the Cochrane Library, EMBASE, and Google Scholar until April 23, 2023, to obtain relevant articles for this study. Our analysis examined several clinical outcomes, including overall survival (OS), progression-free survival (PFS), objective response rate (ORR), and disease control rate (DCR).

**Results:**

In this analysis, a total of 17 articles with 2883 patients were included. Our pooled results indicated that patients with high PNI levels had longer OS (HR: 0.530, 95% CI: 0.456-0.616, *p* < 0.001) and PFS (HR: 0.740, 95% CI: 0.649-0.844, *p* < 0.001), as well as higher ORR (OR: 1.622, 95% CI: 1.251-2.103, *p* < 0.004) and DCR (OR: 1.846, 95% CI: 1.428-2.388, *p* < 0.001). Subgroup analysis showed that PNI cutoff values of 40 to 45 showed greater predictive potential. Subgroup analysis also confirmed that the above findings still hold true in patients with esophageal cancer, gastric cancer, and hepatocellular carcinomas.

**Conclusion:**

The PNI were reliable predictors of outcomes in GIC patients treated with ICIs.

## 1 Introduction

Approximately one-fourth of all cancer cases and one-third of all cancer-related deaths worldwide can be attributed to gastrointestinal cancers (GIC) (1). Systemic therapy remains the cornerstone of treatment for patients with locally advanced or metastatic GIC (2). However, there is a critical need for strategies to reduce metastasis and improve survival. The introduction of immune checkpoint inhibitors (ICIs) over the past five years has resulted in significant advances in the treatment of advanced GIC patients, achieving durable antitumor immune responses and improvements in overall survival (OS) (3–5). But because of the low response rate, scientists are looking for new potential biomarkers that can predict treatment outcomes (3, 6). The identification of well-characterized and predictive biomarkers would facilitate personalized treatment selection based on the anticipated efficacy of therapy and avoid the cost of ineffective treatment.

Numerous studies have demonstrated the relationship between nutritional status and cancer prognosis (7–11). Patients with GIC are particularly affected by nutritional status due to their anatomical features (12). The prognostic nutrition index (PNI) is an index that utilizes the levels of serum albumin and peripheral blood lymphocyte count, initially created to forecast the probability of postoperative complications in surgical patients by evaluating their nutritional status before the operation (13). Recent studies have demonstrated the high accuracy of PNI in predicting treatment outcomes for various cancers, especially GIC (14, 15). Immune system function plays a critical role in the efficacy of ICIs, and the levels of serum albumin and lymphocytes are significant indicators of immune system function.

Notably, the association between PNI levels and the prognosis of GIC patients treated with ICIs remains controversial, and no meta-analysis has been conducted to date. Hence, the aim of this study was to systematically evaluate the predictive value of PNI in ICI-treated GIC patients. The findings of this study can aid in developing effective treatment strategies that facilitate the administration of precise, cost-effective treatments with minimal adverse effects.

## 2 Methods

### 2.1 Literature search strategies

The analysis performed in this study was conducted following the guidelines of the PRISMA statement (16). On April 23, 2023, a comprehensive literature search was carried out using PubMed, EMBASE, and the Cochrane Library. Various search terms, including MeSH terms and keywords, were used to retrieve relevant studies, such as “Immune Checkpoint Inhibitors [MeSH]”, “PD-1 Inhibitors”, “PD-L1 Inhibitors”, “CTLA-4 Inhibitors”, “Pembrolizumab”, “Nivolumab”, “Atezolizumab”, “Ipilimumab”, “Avelumab”, “Tremelimumab”, “Durvalumab”, “Cemiplimab”, “Prognostic Nutritional Index”, “PNI”. Search restricted to English literature. A detailed description of the search strategies is provided in **Table S1**. Additionally, gray literature was searched using Google Scholar, and the reference lists of eligible studies were screened manually.

### 2.2 Inclusion and exclusion criteria

In our study, we strictly included research articles that met the following criteria: patients who were diagnosed with GIC underwent treatment with ICIs, and the prognostic value of PNI was evaluated. Furthermore, these articles reported on at least one of the following outcomes: overall survival (OS), progression-free survival (PFS), objective response rate (ORR), and disease control rate (DCR). We excluded conference abstracts, comments, and case reports from our analysis. In situations where studies had overlapping patients, we prioritized those with the most comprehensive data and robust methodology (3).

### 2.3 Data extraction and quality assessment

In this study, we gathered diverse information from the chosen articles, including the names of the authors, year of publication, duration and location of the study, drugs used for treatment, cancer type, sample size, patient age and gender, and relevant cut-off values and outcomes. We placed greater emphasis on obtaining data from multivariate analyses of hazard ratios (HR) compared to univariate analyses. We also employed the Newcastle-Ottawa Scale (NOS) to appraise the quality of observational studies and classified those with a NOS score of 6 or above as high-quality literature (17). All of the above steps were completed and cross-checked independently by two authors, with decisions sought from the corresponding author on points of dispute.

### 2.4 Statistical methods

Statistical analyses were performed using Stata 15.0 software. To assess heterogeneity, we utilized the chi-squared test. We employed a random effects model when the *p*-value < 0.1 or the I^2^ statistic was > 50%, and a fixed effects model otherwise. We estimated publication bias using both Egger’s and Begg’s tests, and if bias was detected, we utilized the “trim and fill” method to evaluate the influence of the bias on the pooled results. Furthermore, we conducted a sensitivity analysis by excluding each study independently to assess the robustness of the results. *p* < 0.05 was considered statistically significant.

## 3 Results

### 3.1 Characteristics of studies

**Figure 1:**
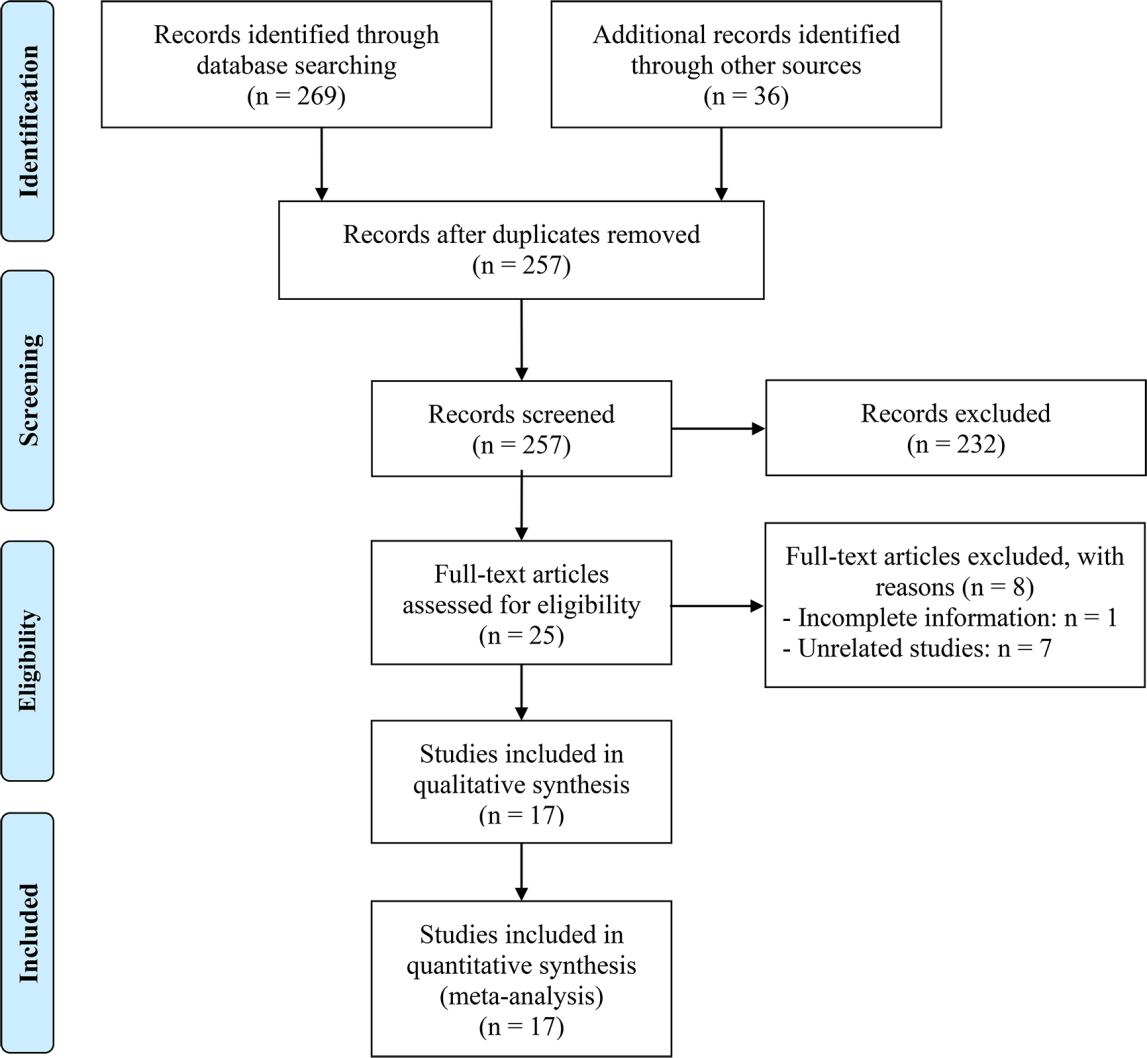
The flow diagram for identifying eligible studies.

After excluding duplicates and screening titles and abstracts, we identified 25 articles for full-text evaluation, among which 17 met the eligibility criteria, resulting in a total of 2883 patients (18–34). The PRISMA flowchart in **Figure 1** depicts the study selection process. **Table 1** provides a comprehensive overview of the characteristics of the eligible studies. We assessed the risk of bias using the Newcastle-Ottawa Scale (NOS), with scores ranging from 6 to 8, indicating a low risk of bias in all included studies. Of the 17 studies, 16 were retrospective, and one was prospective. Four studies were in esophageal squamous cell carcinoma (ESCC) patients; five studies were in gastric cancer (GC) patients; and four studies were in hepatocellular carcinoma (HCC) patients. In addition, there is one study on esophageal cancer (EC) patients, one study on intrahepatic cholangiocarcinoma (ICC) patients, one study on upper gastrointestinal cancer (UGIC) patients and one study on biliary tract cancers (BTC) patients.

**Table 1:** Main characteristics of the studies included.

Study	Study design	Study period	Study region	ICI treatment	Cancer Type	Sample size	Age	Gender (male/female)	Cut-off	Outcome
Chen et al., 2023 (19)	R	08/2019-08/2021	China	Pembrolizumab, Camrelizumab, Sintilimab, Tislelizumab	ESCC	54	67 (43-78)^a^	43/11	45.2	PFS
Ikoma et al., 2023 (20)	R	01/2017-06/2021	Japan	Nivolumab	ESCC	93	70 (38-80)^a^	72/21	48.4	OS
Persano et al., 2023 (27)	R	10/2018-04/2022	Italy, Germany, Portugal, Japan, Korea	Atezolizumab plus Bevacizumab	HCC	773	72 (27-94)^a^	662/111	41.0	OS, PFS
Qi et al., 2023 (28)	P	03/2019-03/2022	China	Pembrolizumab	ESCC	51	62 (39-75)^a^	44/7	52.4	PFS
Tada et al., 2023 (30)	R	09/2020-05/2022	Japan	Atezolizumab plus Bevacizumab	HCC	485	74 (68-80)^a^	389/96	47.0	OS, PFS, ORR, DCR
Wu et al., 2023 (32)	R	09/2018-05/2022	China	Camrelizumab, Pembrolizumab, Nivolumab, Sintilimab, Tislelizumab, Toripalimab	EC	78	58 (46–87)^a^	65/13	40.6	OS, PFS
Yang et al., 2023 (33)	R	03/2017-04/2022	China	Camrelizumab, Sintilimab, Nivolumab, Pembrolizumab	BTC	31	61.0 ± 11.8^c^	19/12	44.3	OS, PFS, ORR, DCR
Book et al., 2022 (18)	R	10/2017-12/2021	Japan	Nivolumab, Pembrolizumab	UGIC	61	71 (46-86)^a^	49/12	-^d^	OS, PFS, DCR
Kim et al., 2022 (21)	R	2015-2019	Korea	Nivolumab, Pembrolizumab	ESCC	60	68 (52–76)^a^	56/4	35.9	OS, PFS, DCR
Lee et al., 2022 (22)	R	10/2017-02/2021	Korea	Nivolumab	GC	35	55 (25-71)^a^	19/16	40.0	OS, PFS
Morelli et al., 2022 (24)	R	06/2014-12/2018	United Kingdom	Pembrolizumab, Nivolumab, Avelumab	GC	57	61 (29-85)^a^	43/14	33.0	OS
Sun et al., 2022 (29)	R	08/2016-12/2020	China	ICIs	GC	89	-	-	44.6	OS, PFS
Yang et al., 2022 (34)	R	02/2019-02/2021	China	Nivolumab, Pembrolizumab, Toripalimab, Camrelizumab, Sintilimab	ICC	73	57 (31-75)^a^	49/24	49.0	OS
Mei et al., 2021 (23)	R	07/2018-12/2019	China	Nivolumab, Pembrolizumab, Toripalimab, Sintilimab, Camrelizumab	HCC	442	52 (21-75)^a^	382/60	48.0	OS, ORR, DCR
Muhammed et al., 2021 (25)	R	2015-2018	Europe, North, America, Asia	ICIs	HCC	362	65 (15-87)^a^	284/78	45.0	OS, PFS, ORR, DCR
Watanabe et al., 2021 (31)	R	10/2015-12/2019	Japan	Nivolumab	GC	110	71/39^b^	79/31	40.0	OS, PFS, ORR, DCR
Namikawa et al., 2020 (26)	R	10/2017-12/2019	Japan	Nivolumab	GC	29	71 (49–86)^a^	19/10	31.1	OS, PFS

^a^medians (ranges); ^b^≥ 65 vs. < 65; ^c^mean ± standard deviation; ^d^ESCC patients with cut-off = 42.8 and GC patients with cut-off = 37.2; R, retrospective study; P, prospective study; OS, overall survival; PFS, progression-free survival; ORR, objective response rate; DCR, disease control rate; ESCC, esophageal squamous cell carcinoma; EC, esophageal cancer; GC, gastric cancer; HCC, hepatocellular carcinoma; BTC, biliary tract cancers; ICC, intrahepatic cholangiocarcinoma; UGIC, upper gastrointestinal cancer; ICIs, immune checkpoint inhibitors.

### 3.2 Baseline PNI levels and OS

**Figure 2:**
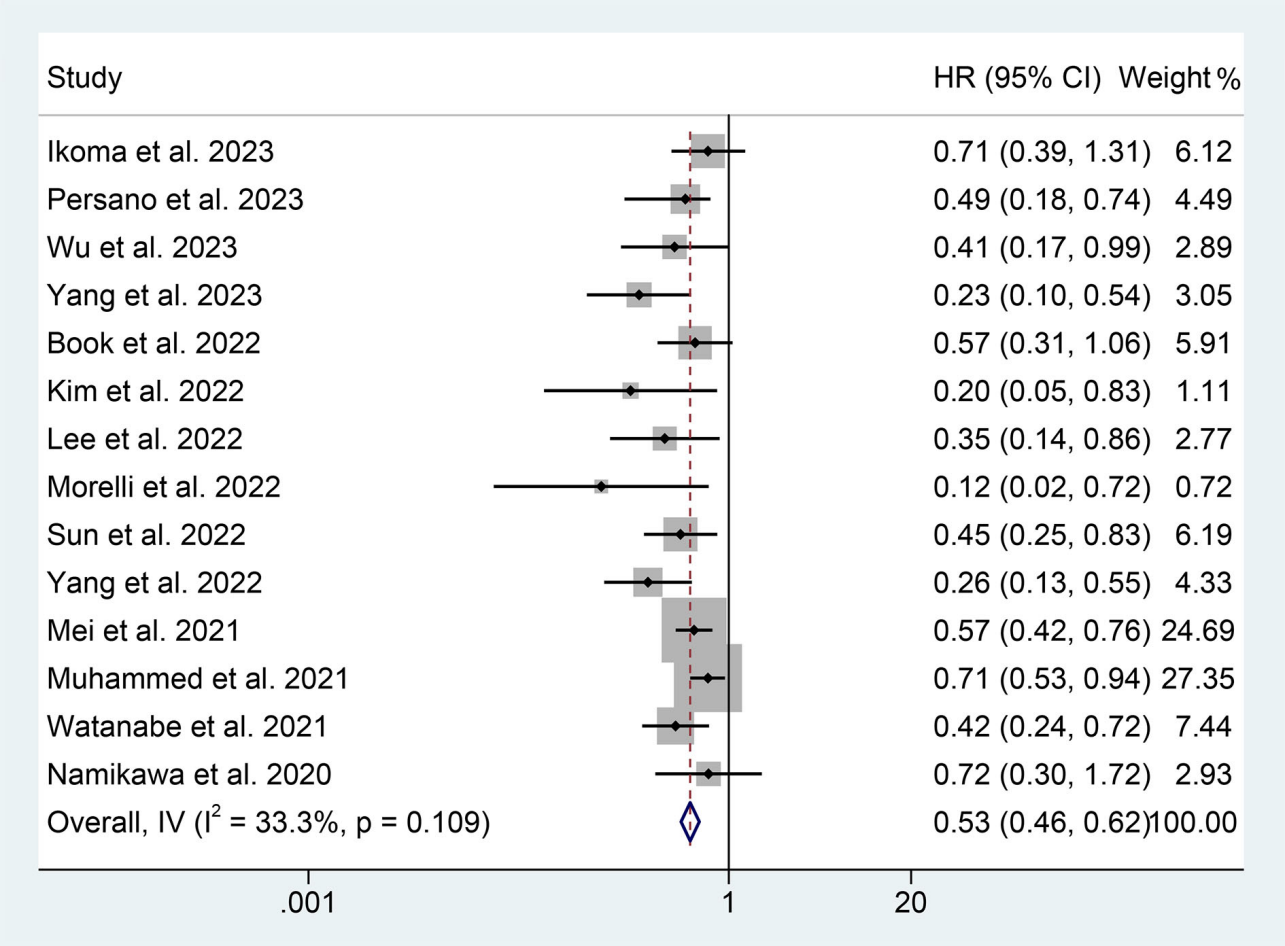
Forest plots of the relationship between prognostic nutritional index and overall survival. HR, hazard ratio; CL, confidence interval.

Through the analysis of data from 14 studies with 2293 patients, we aimed to explore the correlation between PNI levels and OS in ICI-treated GIC patients. Considering no significant heterogeneity across the studies (I^2^ = 33.2%, *p* = 0.109), we employed a fixed-effects model to estimate the pooled HR. The results revealed that high PNI levels were significantly related to longer OS (HR: 0.530, 95% CI: 0.456-0.616, *p* < 0.001), as depicted in **Figure 2**.

**Figure 3:**
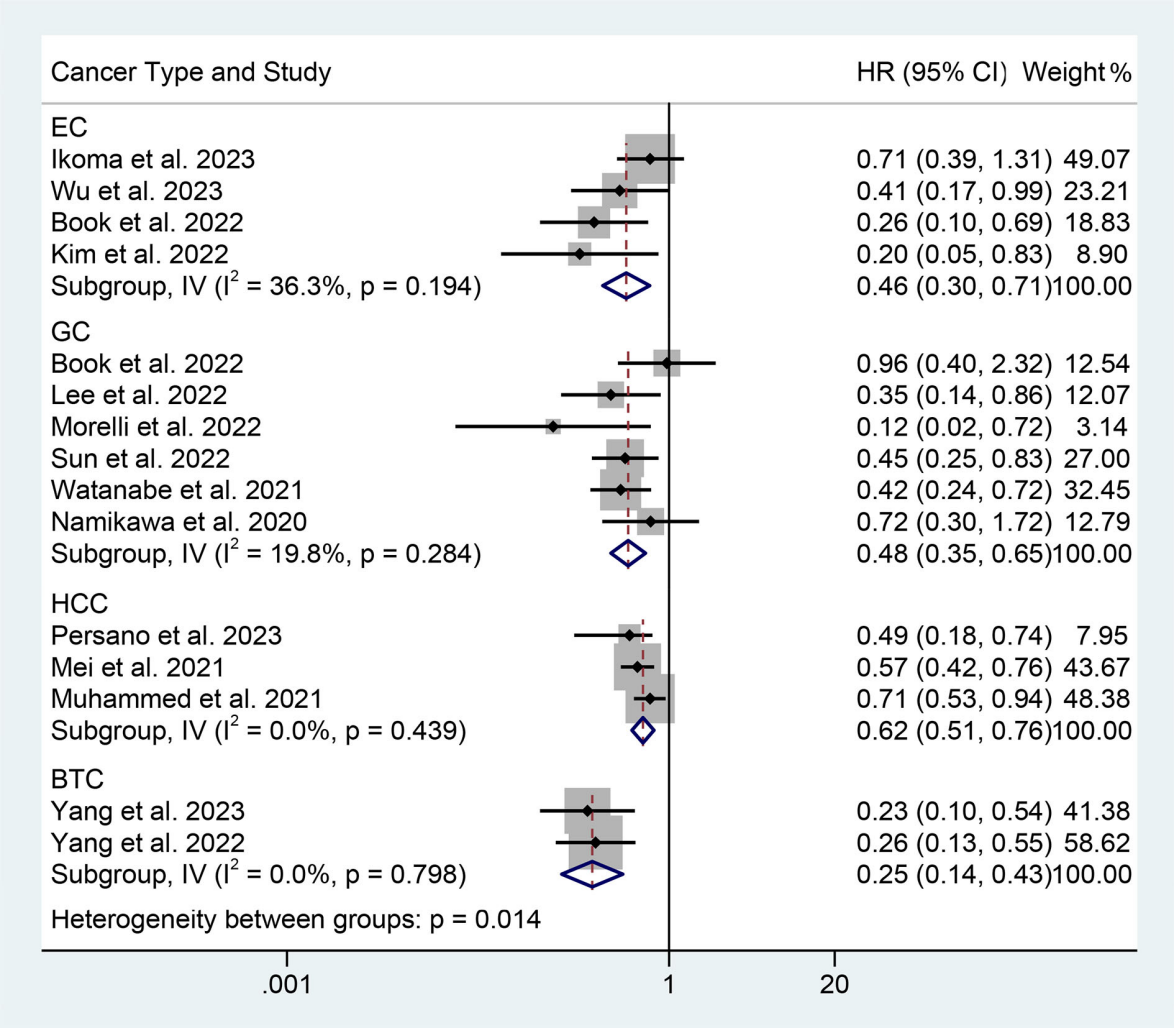
Subgroup analysis of the relationship between prognostic nutritional index and overall survival based on the cancer type. HR, hazard ratio; CL, confidence interval.

**Figure 4:**
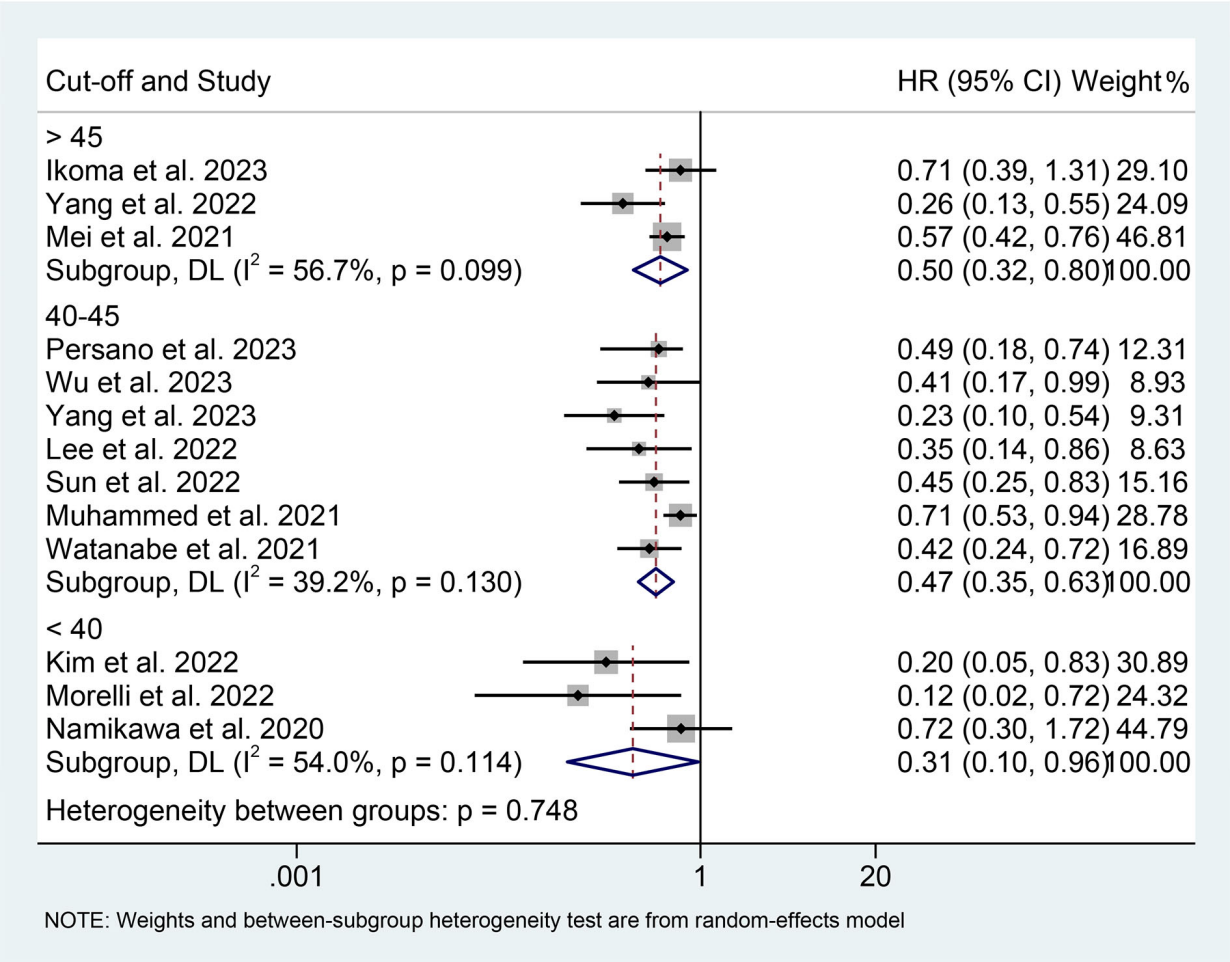
Subgroup analysis of the relationship between prognostic nutritional index and overall survival based on the cut-off. HR, hazard ratio; CL, confidence interval.

We performed a subgroup analysis based on cancer type, cut-off values, and the Cox model. We found that a high PNI was associated with a better prognosis in patients with EC, GC, HCC, or BTC (**Figure 3**). Differences in PNI cutoff values do not affect the correlation between PNI and OS in ICI-treated GIC patients (**Figure 4**). Both univariate and multivariate analyses confirmed the above findings (**Figure S1**).

### 3.3 Baseline PNI levels and PFS

**Figure 5:**
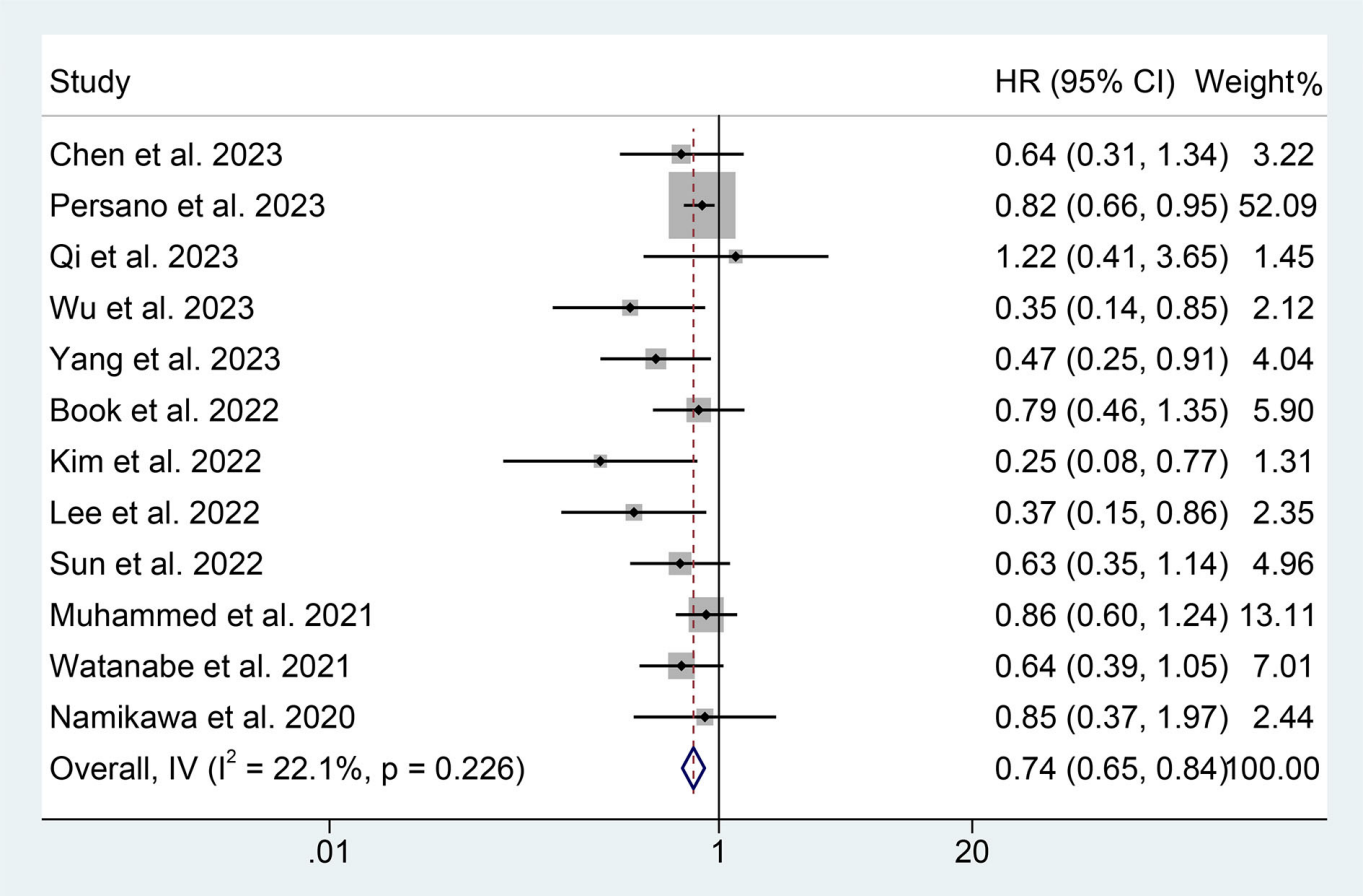
Forest plots of the relationship between prognostic nutritional index and progression-free survival. HR, hazard ratio; CL, confidence interval.

We also investigate the correlation between PNI and PFS in GIC patients treated with ICIs using data from 12 studies with 1733 patients. We demonstrated that patients with high PNI levels had a lower risk of progression (HR: 0.740, 95% CI: 0.649-0.844, *p* < 0.001, **Figure 5**). No significant heterogeneity was observed (I^2^ = 22.1%, *p* = 0.226); we used a fixed-effects model for our analysis.

**Figure 6:**
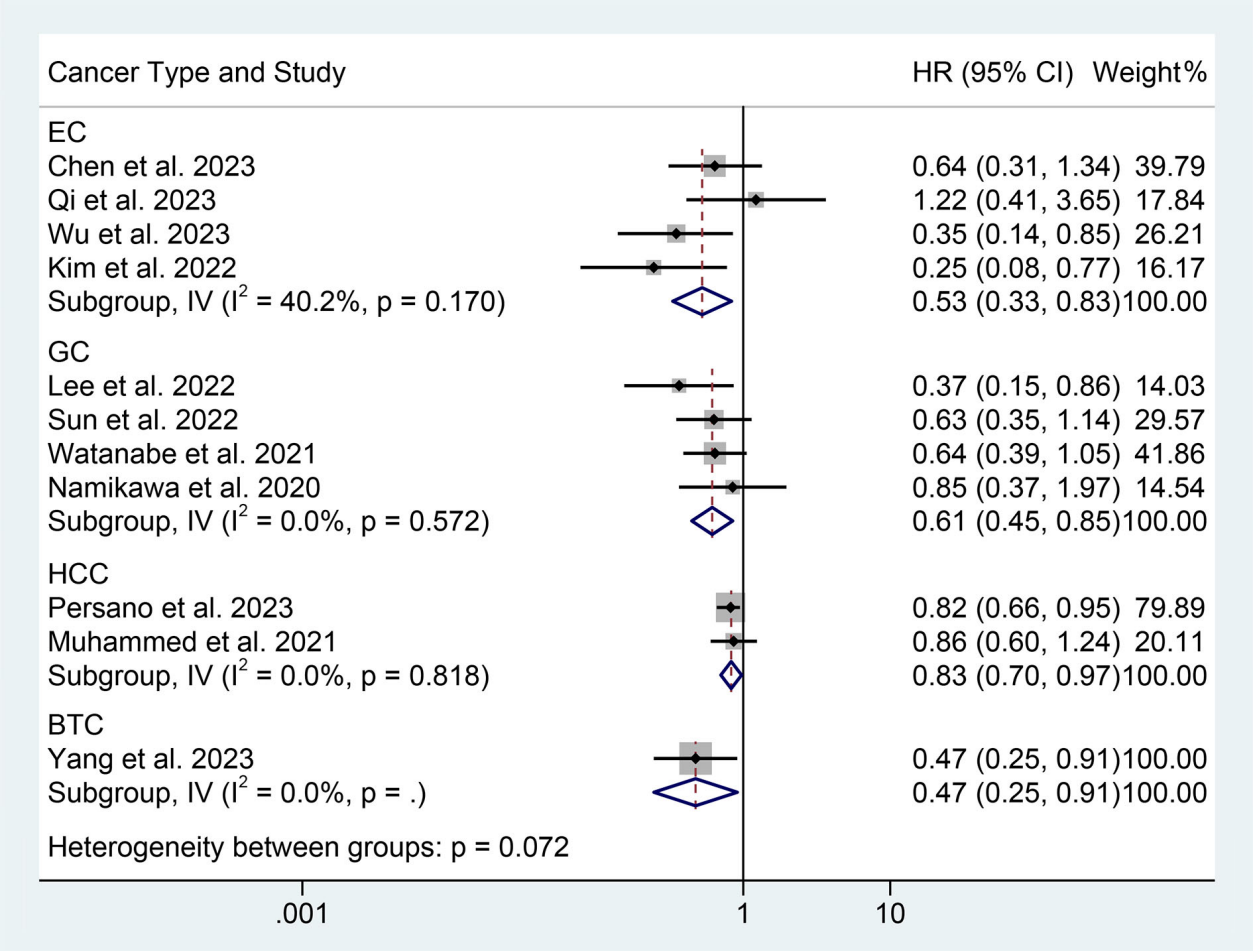
Subgroup analysis of the relationship between prognostic nutritional index and progression-free survival based on the cancer type. HR, hazard ratio; CL, confidence interval.

**Figure 7:**
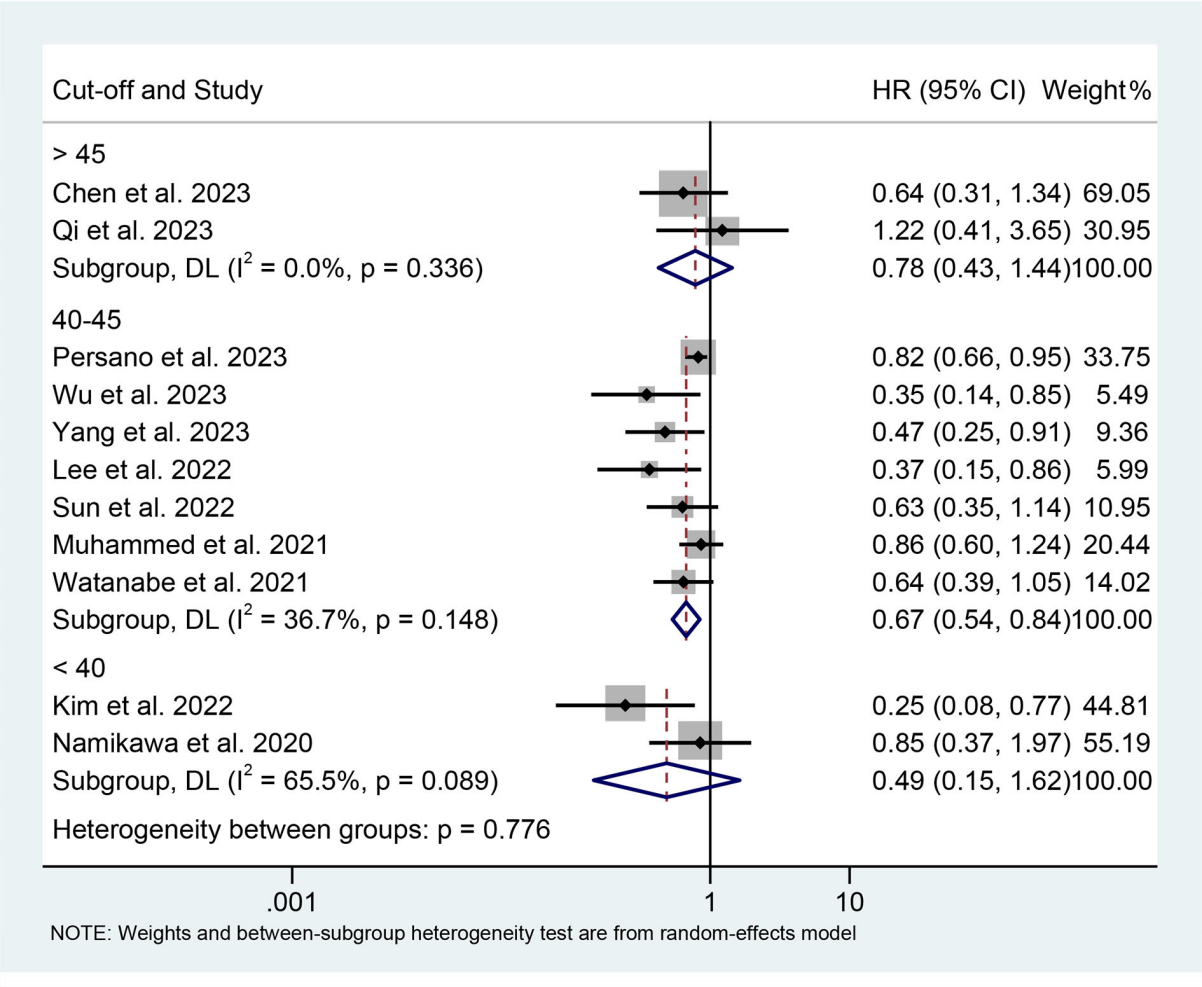
Subgroup analysis of the relationship between prognostic nutritional index and progression-free survival based on the cut-off. HR, hazard ratio; CL, confidence interval.

The results of the subgroup analysis showed that the relationship between high PNI levels and longer PFS was consistent in GIC patients with EC, GC, and HCC (**Figure 6**). Notably, PNI predicted PFS in patients with GIC only when the cut-off value was between 40 and 45 (**Figure 7**).

The findings of the multivariate analysis also support these findings; although the univariate analysis revealed that PNI was not associated with patient PFS, we consider the conclusions drawn from the former to be more reliable due to its more methodologically rigorous nature and larger number of inclusions (**Figure S2**).

### 3.4 Baseline PNI levels and ORR and DCR

**Figure 8:**
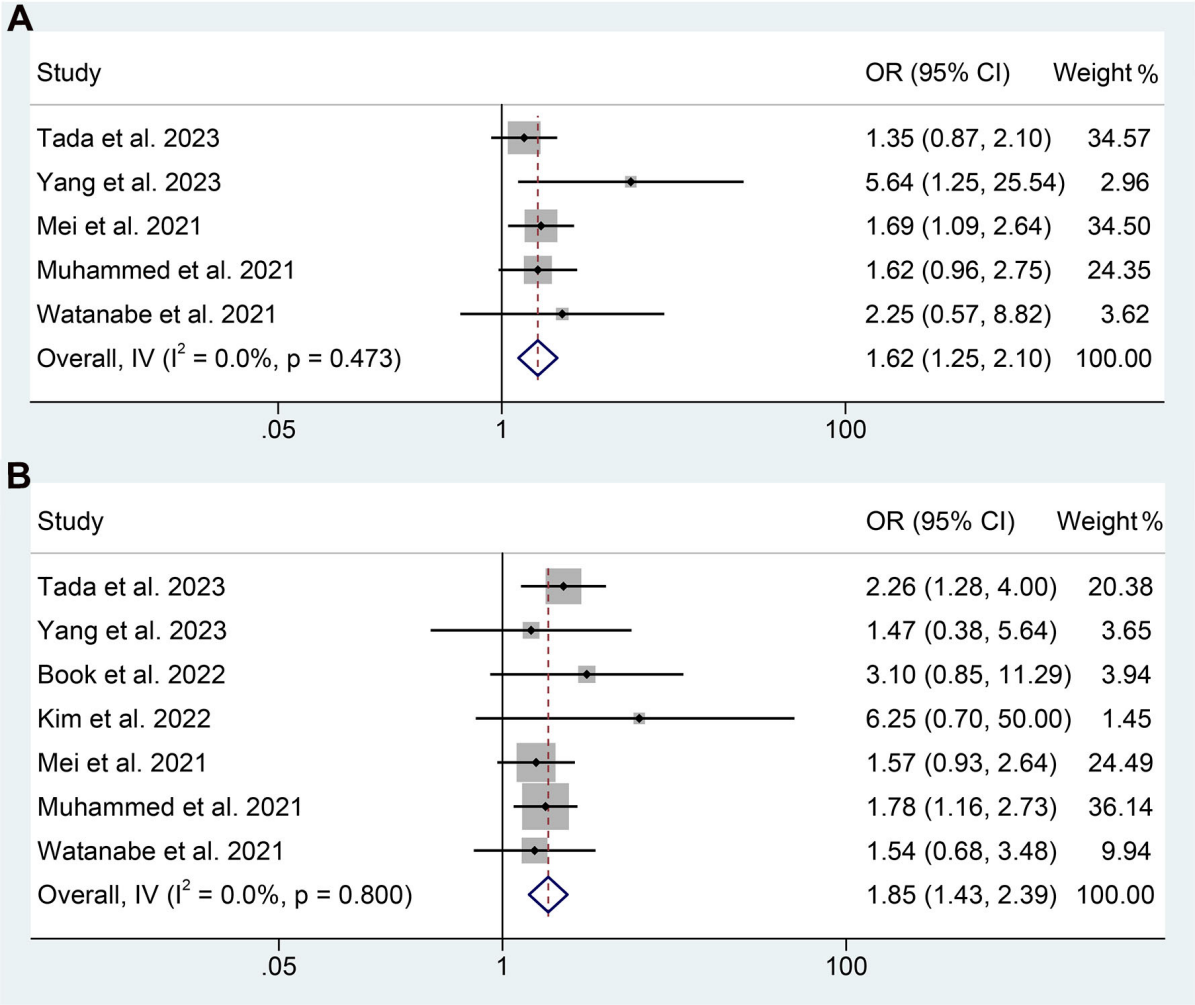
Forest plots of the relationship between prognostic nutritional index and objective response rate **(A)** and disease control rate **(B)**. OR, odds ratio; CL, confidence interval.

Subsequently, we conducted an analysis to investigate the correlation between PNI levels and response to ICI therapy in GIC patients. The presence of notable heterogeneity was not observed in the results presented in **Figures 8A, B**, and hence a fixed-effect model was implemented. Our findings revealed that GIC patients with high PNI levels had a higher ORR (5 studies with 1430 patients, OR: 1.622, 95% CI: 1.251-2.103, *p* < 0.004, **Figure 8A**) and DCR (7 studies with 1551 patients, OR: 1.846, 95% CI: 1.428-2.388, *p* < 0.001, **Figure 8B**).

### 3.5 Sensitivity analysis

**Figure 9:**
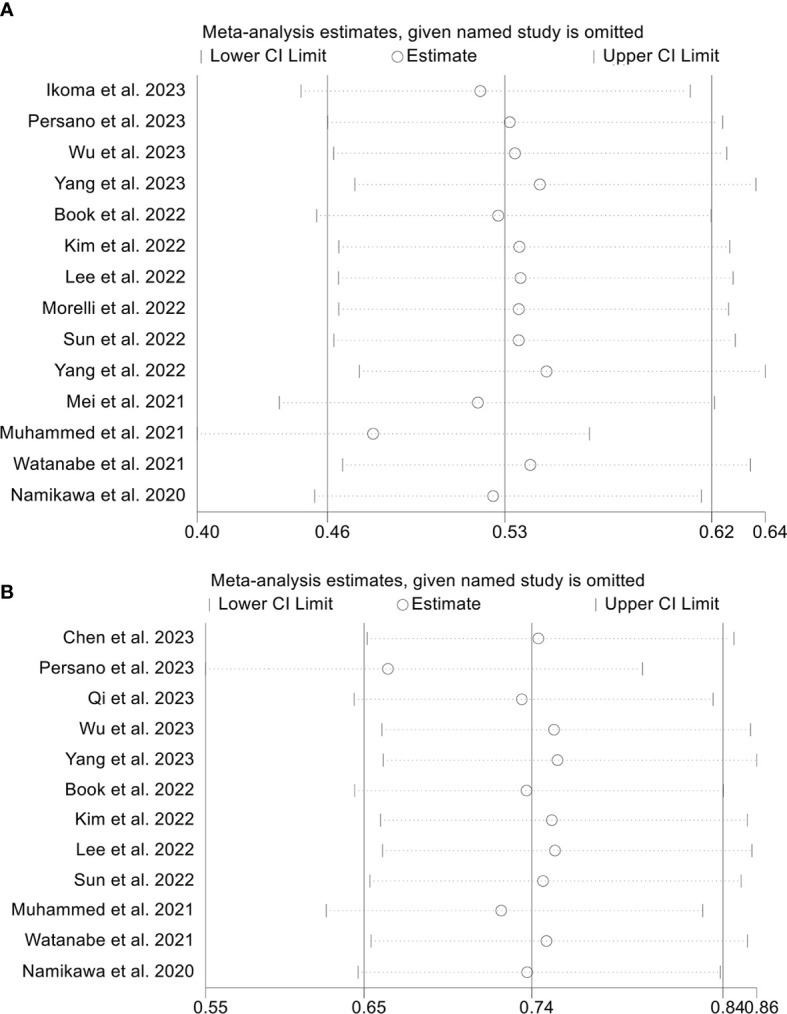
Sensitivity analysis of the association between prognostic nutritional index and overall survival **(A)** and progression-free survival **(B)**. CL, confidence interval.

**Figure 10:**
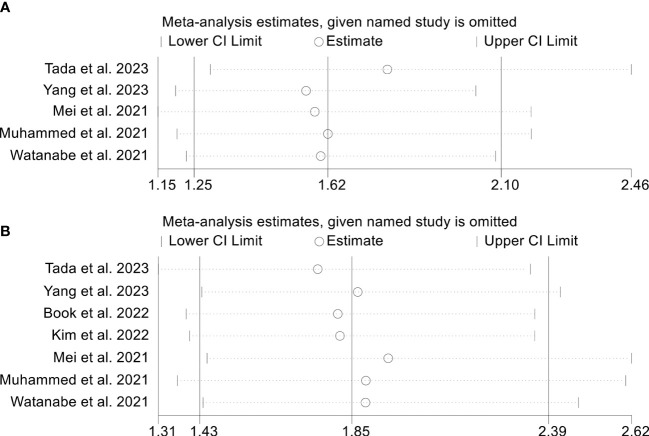
Sensitivity analysis of the association between prognostic nutritional index and objective response rate **(A)** and disease control rate **(B)**. CL, confidence interval.

To assess the robustness of the findings, a sensitivity analysis was conducted by iteratively excluding each study and examining the impact on the overall results. Our analysis indicated that the exclusion of any individual study did not significantly affect the pooled HR for OS. Specifically, the HR estimates for OS ranged from 0.474 (95% CI: 0.398-0.566) when excluding Muhammed et al., 2021 (25) to 0.547 (95% CI: 0.469-0.637) when excluding Yang et al., 2022 (34), as depicted in **Figure 9A**. Furthermore, the sensitivity analysis demonstrated that the removal of any individual study did not significantly impact the overall results for PFS. The range of HR values varied from 0.662 (95% CI: 0.547-0.800) after excluding Persano et al., 2023 (27) to 0.754 (95% CI: 0.659-0.862) after excluding Yang et al., 2022 (33) (**Figure 9B**). Similarly, sensitivity analysis showed that removing any of the studies did not affect the ORR and DCR results (**Figures 10A, B**).

### 3.6 Publication bias

**Figure 11:**
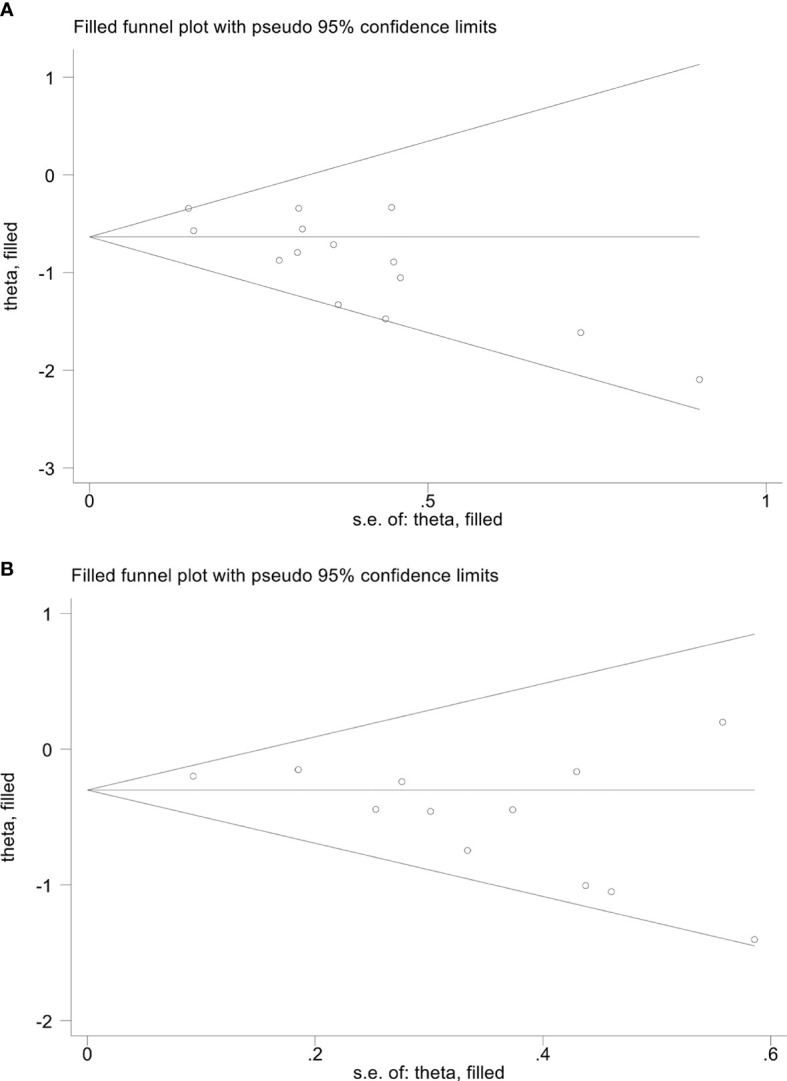
The picture of the trim-and-fill method terms of overall survival **(A)** and progression-free survival **(B)**. Theta, the effect estimate; S.e. of: theta, the corresponding standard error.

We performed Begg’s and Egger’s tests to evaluate the potential publication bias in our meta-analysis. The findings indicated no considerable publication bias for ORR (Egger’s test: *p* = 0.241, Begg’s test: *p* = 0.230) and DCR (Egger’s test: *p* = 0.086, Begg’s test: *p* = 0.081). Nevertheless, we detected publication bias in OS (Egger’s test: *p* = 0.003, Begg’s test: *p* = 0.021) and PFS (Egger’s test: *p* = 0.027, Begg’s test: *p* = 0.064) based on Egger’s test. To address this issue, we utilized the trim and fill method to estimate the number of potential missing studies in OS. The results showed no change in pooled HR without the missing study being incorporated (**Figure 11**).

## 4 Discussion

Our investigation aimed to explore the prognostic significance of PNI in GIC patients receiving ICI therapy. Through a meta-analysis of relevant studies, we established a strong association between elevated PNI levels and favorable OS and PFS and higher ORR and DCR. Furthermore, subgroup analysis showed that PNI cutoff values of 40 to 45 showed greater predictive potential.

While ICIs have emerged as a promising treatment for GIC patients, the factors affecting their efficacy remain unclear. Multiple biomarkers have been proposed for predicting response to ICIs, including tumor mutation burden, microsatellite instability/mismatch repair deficiency, tertiary lymphoid structures, and tumor-infiltrating lymphocytes (35). However, the application of these biomarkers in clinical practice is limited by challenges such as immature detection technology, difficulty obtaining specimens, and high costs.

PNI is calculated as 5 × peripheral lymphocyte count (10^9^/L) +serum albumin (g/L), which includes albumin and lymphocyte count, reflecting nutritional and immune status, respectively. In upper gastrointestinal cancer patients, nutritional problems are prevalent in up to 90% of cases, mainly due to reduced food intake and increased nutrition consumption by tumors (36). Long-standing research has linked malnutrition to a worse tumor prognosis (37, 38). While low albumin levels are indicative of malnutrition, they can also serve as a biomarker for systemic inflammation (39). Inflammatory factors have been shown to inhibit albumin synthesis, while oxidative stress can result in albumin denaturation, contributing to a rapid decrease in serum albumin levels in patients with an inflammatory state (40, 41). Another crucial element in the development of tumors is the tumor microenvironment. Through the attraction of T lymphocytes, tumor-associated macrophages, and circulating cytokines, inflammatory factors can significantly impact tumor cell proliferation, angiogenesis, and tumor invasion/metastasis (42, 43). A crucial component of adaptive immunity is the lymphocyte. The immune system’s capacity to prevent tumor cell growth and metastasis may decline when lymphocyte numbers drop, hastening the development of tumors (44, 45). Therefore, the combination of albumin and lymphocyte counts in the PNI can provide a more comprehensive reflection of the host condition.

Initially used to evaluate the immunotrophic status and surgical risks of gastrointestinal surgery patients, PNI has since been applied to other cancer types, including EC (46), GC (26), HCC (47), and pancreatic cancer (48). The success of ICIs in combating tumors is attributed to their ability to alleviate the suppression of tumor cells by the immune system. Hence, the nutritional and immune statuses of patients are critical determinants of the efficacy of ICIs (49). In this study, we performed the first meta-analysis to confirm that PNI predicted the response of GIC patients to ICI therapy. PNI possesses several benefits that make it convenient for daily clinical practice. It is readily available, easily quantifiable, repeatable, and relatively cost-effective to assess (50). As a result, due to its well-established impact on the host’s nutritional and immune status as well as cancer, the PNI could serve as a useful tool in predicting the therapeutic outcomes of ICIs in GIC patients. Individualized and timely nutritional and immunological interventions may improve the prognosis of patients with low baseline PNI.

It is noteworthy that the majority of studies included in this analysis were retrospective cohort studies, which may limit their statistical validity. In addition, the types of ICIs used in each study are not entirely consistent. Therefore, it is imperative to perform additional high-quality investigations with larger sample sizes, particularly multicenter prospective studies, to corroborate and refine our findings.

## Data availability statement

The original contributions presented in the study are included in the article/**Supplementary Material**. Further inquiries can be directed to the corresponding authors.

## Author contributions

LZ, WM, BH, and WW conceived and designed the study. LZ, WM, ZQ, TK, and KW were responsible for the collection and assembly of data, data analysis, and interpretation. LZ, ZQ, and WM were involved in writing the manuscript. LZ, QZ, WM, BH, and WW revised the manuscript. All authors contributed to the article and approved the submitted version.

## Supplementary material

Supplementary Table 1 The detailed search strategies for Pubmed.

Supplementary Figure 1 Subgroup analysis of the relationship between prognostic nutritional index and overall survival based on the Cox model. HR, hazard ratio; CL, confidence interval.

Supplementary Figure 2 Subgroup analysis of the relationship between prognostic nutritional index and progression-free survival based on the Cox model. HR, hazard ratio; CL, confidence interval.
