# Supplementary figures and images for: Prognostic nutritional index as a prognostic biomarker for gastrointestinal cancer patients treated with immune checkpoint inhibitors
Source: Front Immunol. 2023 Jul 21;14:1219929. doi: 10.3389/fimmu.2023.1219929 (PMC10401046; doi:10.3389/fimmu.2023.1219929)

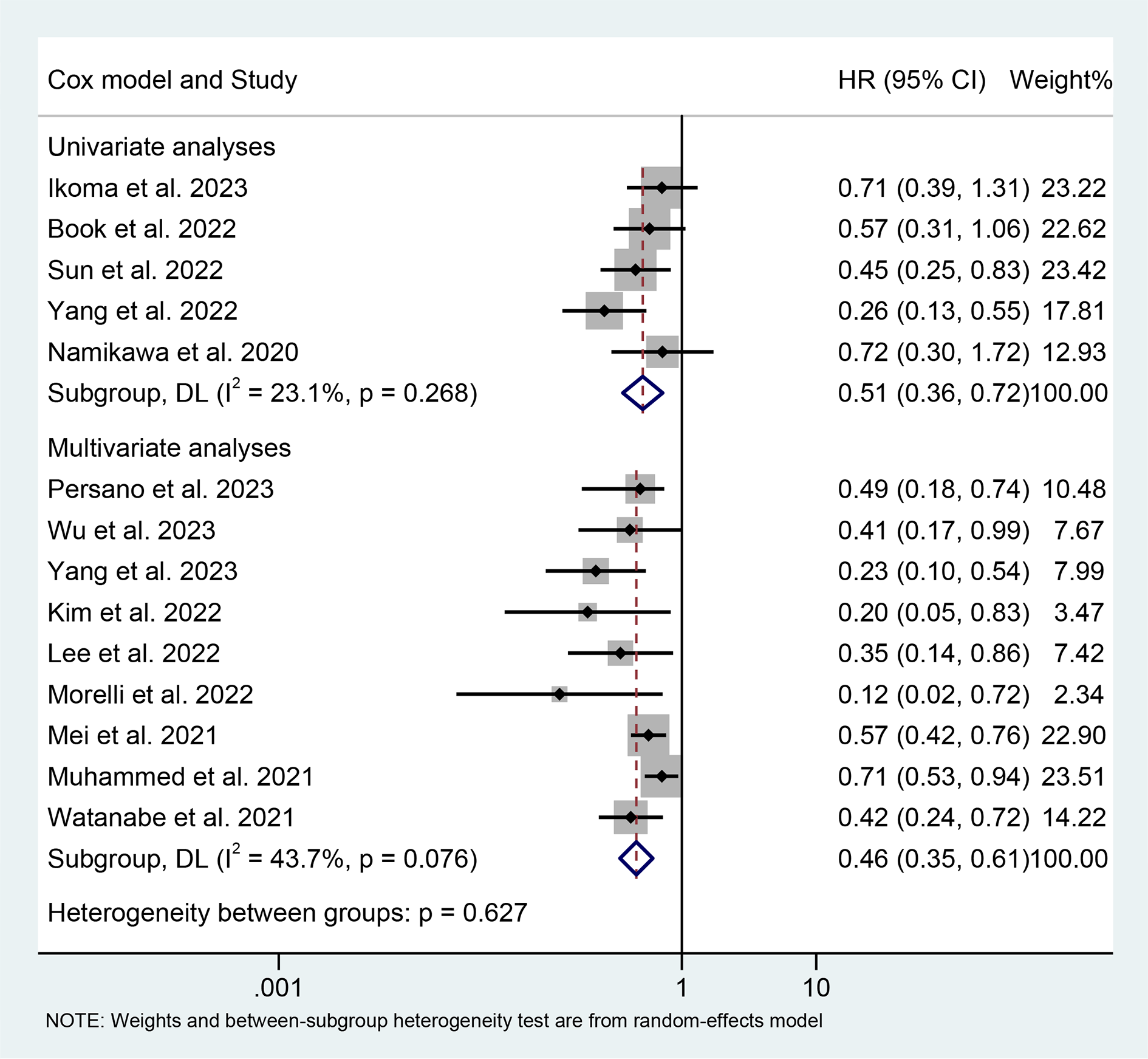

Supplement: Supplementary Figure 1 — Subgroup analysis of the relationship between prognostic nutritional index and overall survival based on the Cox model. HR, hazard ratio; CL, confidence interval. [file Image_1.tif]

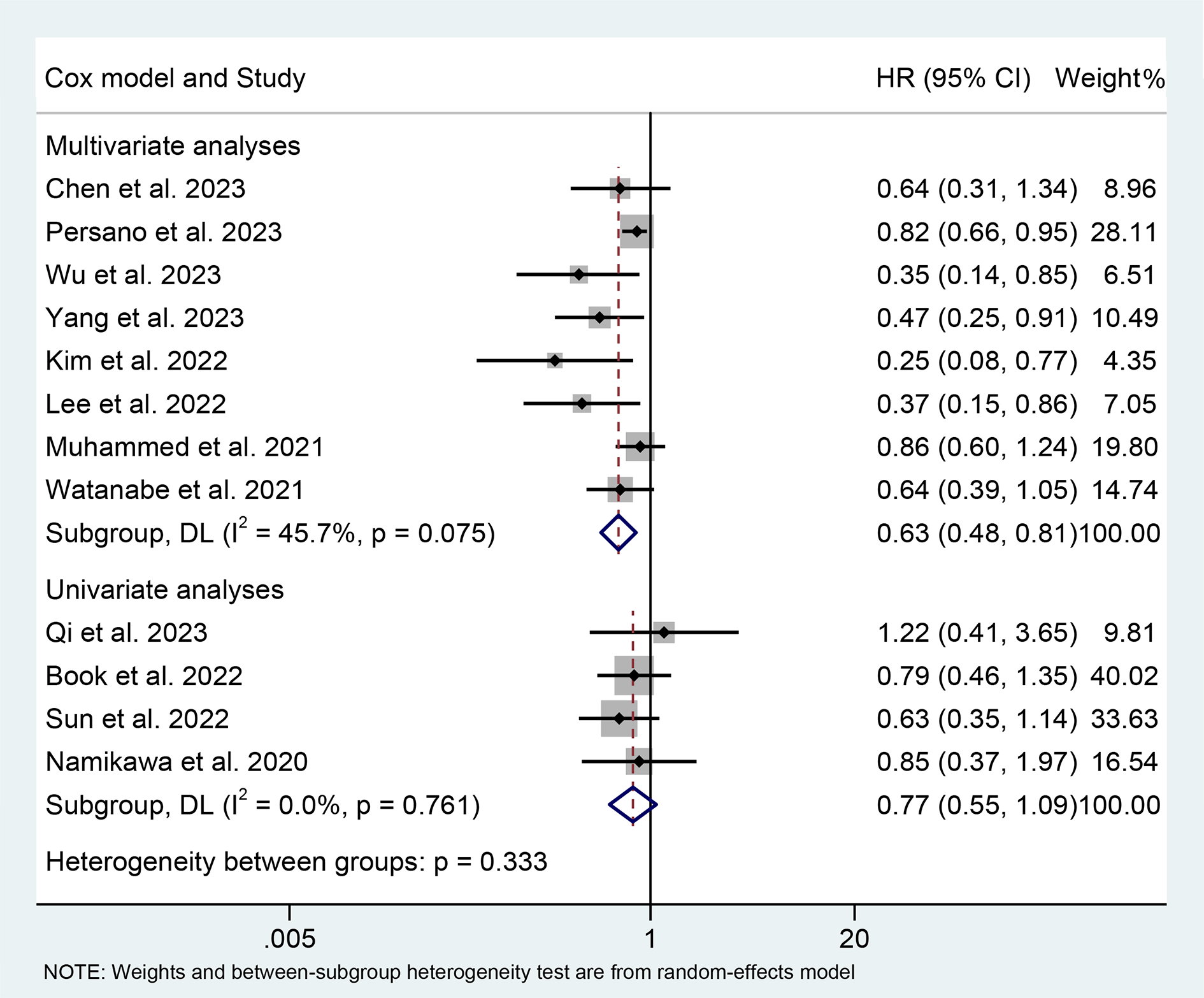

Supplement: Supplementary Figure 2 — Subgroup analysis of the relationship between prognostic nutritional index and progression-free survival based on the Cox model. HR, hazard ratio; CL, confidence interval. [file Image_2.tif]
